# ERK MAP Kinase Activation in Spinal Cord Regulates Phosphorylation of Cdk5 at Serine 159 and Contributes to Peripheral Inflammation Induced Pain/Hypersensitivity

**DOI:** 10.1371/journal.pone.0087788

**Published:** 2014-01-31

**Authors:** Xiaoqin Zhang, Honghai Zhang, Haijun Shao, Qingsheng Xue, Buwei Yu

**Affiliations:** 1 Department of Anesthesia, Rui Jin Hospital, School of Medicine, Shanghai Jiao Tong University, Shanghai, P.R. China; 2 Department of Anesthesia, Hangzhou First People's Hospital, Nanjing Medical University, Zhejiang, P.R. China; Albert Einstein College of Medicine, United States of America

## Abstract

Cyclin-dependent kinase 5 is a proline-directed serine/threonine kinase and its activity participates in the regulation of nociceptive signaling. Like binding with the activators (P35 or P25), the phosphorylation of Cdk5 plays a critical role in Cdk5 activation. However, it is still unclear whether Cdk5 phosphorylation (p-Cdk5) contributes to pain hyperalgesia. The aim of our current study was to identify the roles of p-Cdk5 and its upstream regulator in response to peripheral inflammation. Complete Freund's adjuvant (CFA) injection induced acute peripheral inflammation and heat hyperalgesia, which was accompanied by sustained increases in phospho-ERK1/2 (p-ERK1/2) and phospho-Cdk5^S159^ (p-Cdk5^S159^) in the spinal cord dorsal horn (SCDH). CFA-induced p-ERK primarily colocalized with p-Cdk5^S159^ in superficial dorsal horn neurons. Levels in p-ERK and p-Cdk5 were also increased in the 2^nd^ phase of hyperalgesia induced by formalin injection, which can produce acute and tonic inflammatory pain. MAP kinase kinase inhibitor U0126 intrathecal delivery significantly suppressed the elevation of p-Cdk5^S159^, Cdk5 activity and pain response behavior (Heat hyperalgesia, Spontaneous flinches) induced by CFA or formalin injection. Cdk5 inhibitor roscovitine intrathecal administration also suppressed CFA-induced heat hyperalgesia and Cdk5 phosphorylation, but did not attenuate ERK activation. All these findings suggested that p-Cdk5^S159^ regulated by ERK pathway activity may be a critical mechanism involved in the activation of Cdk5 in nociceptive spinal neurons contributes to peripheral inflammatory pain hypersensitivity.

## Introduction

Cyclin-dependent kinase 5 (Cdk5) is a member of the cyclin-dependent kinase family [1]. Cdk5 activity plays multiple roles in neuronal development and synaptic plasticity [2], [3], [4], [5]. The activation of Cdk5 requires the presence of its activators, p35 and p39 or their truncated forms p25 and p29 [6], [7], [8]. Further studies suggest that high levels of Cdk5, p35 or p25 expression as well as Cdk5 kinase activity in nociceptive primary afferent neurons contribute to pain sensitization after inflammation. Inhibition or knockout of Cdk5 or p35 attenuates these responses to nociceptive heat stimulus, and overexpression of Cdk5 or p35 potentiates these responses, indicating Cdk5 plays an important role in nociceptive process [9], [10], [11], [12]. As a proline-directed serine/threonine kinase, Cdk5 catalytic activation is not only influenced by the binding of the activators, but also by the phosphorylation [13]. Phosphorylation at the site of serine 159 or tyrosine 15 dramatically increases Cdk5 activation and the Ser159 residue is the major phosphorylation target for Cdk5-activating kinases [13], [14]. Residue Ser159 phosphorylation contributes to the specificity of the Cdk5-p35 interaction [15] and enhances Cdk5/p25 activity [16]. However, it is still unknown whether Cdk5 phosphorylation at Ser159 site (p-Cdk5^S159^) in the spinal cord dorsal horn (SCDH) may contribute to pain sensitization.

Cdk5 activity can regulate many proteins associated with cell morphology, synaptic activity, neuronal survival, and apoptosis [1], [17], but what is responsible for Cdk5 activation? Previous studies have reported that Extracellular signal-regulated kinase 1 and 2 (ERK1/2) activation is implicated in the regulation of Cdk5 activity via induction of p35 protein expression [18], [19], [20]. ERK1/2 pathway is a critical cellular signaling pathway associated with numerous extracellular signals transmitting to intracellular receptors activated by extracellular stimuli [21], [22]. ERK1/2 is associated with the establishment and maintenance of exacerbated nociception and rapidly activated following innocuous and noxious stimulation [23], [24]. Therefore, we determined whether ERK activity could regulate Cdk5^S159^ phosphorylation after peripheral inflammatory stimulation. Interestingly, it has reported that ERK1/2 activity through phosphorylation is also regulated by Cdk5 activity [25] in inflammatory/neuropathic hyperalgesia and allodynia [26], however, a previous study has also shown that Cdk5 inhibitor roscovitine had no effect on ERK1/2 activity in SCDH post peripheral injection of CFA [9]. In this study, we investigated the role of p-Cdk5^S159^ in nociceptive transmission in a CFA or formalin induced inflammation model. Furthermore, to explore the link between the activation of ERK1/2 and p-Cdk5^S159^ in pain signaling, we delivered the MEK (MAP kinase kinase) inhibitor U0126 and Cdk5 inhibitor roscovitine respectively, and then determined the activity of ERK1/2 and p-Cdk5^S159^ expression in the dorsal horn of spinal cord enlargement after CFA injection.

## Materials and Methods

### Animals and drugs

Male Sprague-Dawley rats (220∼250 g) were obtained from Shanghai laboratory animal center (Shanghai, China). All experimental procedures were approved by the Committee of Animal Use for Research and Education of Shanghai Jiao Tong University School of Medicine. The numbers of animals used in all experiments were minimized according to the guidelines established by the Ethical Issues of the International Association for the study of pain. Rats were housed in a climate-controlled environment on a12 h light/dark cycles and free access to food and water. CFA (100 µl, Sigma, St. Louis, MO, USA) or 5% formalin (100 µl) was injected into the plantar surface of the left hind paw of rats. For intrathecal drug delivery, a polyethylene-10 catheter was implanted into the intrathecal space of the spinal cord at the lumbar enlargement, and 5 µl of the MEK inhibitor U0126 (2 µg, Sigma) dissolved in 4% dimethyl sulfoxide (DMSO), was administered 30 min before treatment and once per day (for 7 days) after treatment, DMSO (4%) was injected as vehicle control. 5 µl of the Cdk5 inhibitor roscovitine (100 µg, R-1234, LC Laboratories, Woburn, MA, USA) dissolved in 10% DMSO, was administered 30 min before treatment and once per day (for 7 days) after treatment, DMSO (10%) was injected as vehicle control.

### Radiant heat paw withdrawal test

Thermal escape latencies were evaluated using a radiant heat stimulation apparatus as previously described [29]. Briefly, individual rat was placed into a plastic chamber with a glass floor (about 1 mm thickness) allowed to acclimate to their environment for 30 min before testing. A focused projection bulb positioned under the glass floor directly beneath the hind paw. Paw withdrawal latencies (PWLs) in response to radiant heat (30°C) were recorded. To avoid burning the skin of rats, the exposure cut-off time was limited to 20 s. The PWL of each rat was measured 3 times at intervals of 5 min and assessed at 2 h, 6 h, 1 d, 3 d and 7 d post CFA injection.

### Formalin-induced spontaneous flinches

To measure spontaneous flinches for formalin injection, rat was acclimated in behavior chamber (30 cm×30 cm) with a plexiglass floor for at least 1 hour. 30 minutes after 2 µg MEK inhibitor U0126 or vehicle (DMSO) injection, 100 µl of 5% formalin solution was then injected subcutaneously into the plantar surface of the left hind paw of rat. Animal was immediately returned to the chamber for observation. Video recorded from below with a webcam (Logitech) for 1 hour. Flinching was characterized as a rapid and brief withdrawing or flexing of the injected paw and quantified by counting the number of flinches in five-minute bins for one hour.

### Inclined plane test

Motor function was assessed by the inclined plane test as described in a previous study [27]. Briefly, after intrathecal injection of 2 µg U0126 or 100 µg roscovitine and before CFA or formalin injection, rat was placed on an inclined plane which can be adjusted to provide a slope of varying grade. The maximum angle of the plane, at which the animal can maintain its position without falling, was recorded and used for statistical analysis.

### Western blot

Rats were deeply anesthetized with sodium pentobarbital (50 mg/kg, i.p.), then the dorsal horns of the L4/5 spinal cords were removed and immediately homogenized in ice-chilled lysis buffer (50 mM Tris, pH 7.4, 150 mM NaCL, 1.5 mM MgCL_2_, 10% glycerol, 1% Triton X-100, 5 mM EGTA, 0.5 mg/ml leupeptin, 1 mM PMSF, 1 mM Na_3_VO_4_, 10 mM NaF, and a protease inhibitor cocktail from Roche). Homogenates were centrifuged at 13,000 g for 15 min at 4°C and protein concentrations determined using the BCA assay kit (Pierce, Rockland, IL). Equal amounts of samples (40 µg) of protein were separated by SDS–PAGE using 10% running gels and transferred to nitrocellulose membranes (Bio-Rad Laboratories). After blocking with 5% non-fat milk in TBST (50 mM Tris–HCl, pH 7.5, 150 mM NaCl, and 0.1% Tween 20) for 1 h at room temperature, the membranes were incubated overnight at 4°C with primary antibody, P-Cdk5^S159^ (1∶1000, Santa cruz, CA, USA); Cdk5 antibody (1∶1000, Millipore, Billerica, MA, USA); p-EPK1/2 (1∶1000, Bioworld, St. Louis Park, MN, USA); ERK1/2 (1∶1000, Bioworld, St. Louis Park, MN, USA); β-actin (1∶20000, Sigma, St. Louis, MO, USA). The membranes were washed in TBST 10 min for three times, and then incubated with HRP-conjugated secondary antibody (1∶2000 dilution, goat anti-rabbit or mouse) for 1 h at room temperature. Finally, the blots were developed with the lightening chemiluminescence kit (Millipore, Billerica, MA, USA).

### Immunofluorescence

Rats were anesthetized with sodium pentobarbital (50 mg/kg, i.p.) and then perfused with 200 ml 0.9% saline followed by 4% ice-cold paraformaldehyde. L4/L5 ipsilateral spinal cords were immediately removed and post-fixed in 4% paraformaldehyde overnight at 4°C, then immersed in 30% sucrose until the samples sank to the bottom of the container. The tissues were embedded with Tissue-Tek (Sakura Finetek, Torrance, CA) and frozen in −80°C. Frozen tissue sections were cut coronally in a cryostat at a thickness of 8 µm. For staining, sections were blocked for 1 h at room temperature with 0.35% Triton X-100/1% BSA. Subsequently, sections were incubated overnight at 4°C with rabbit polyclonal anti-P-Cdk5^S159^ (1∶200, Santa cruz, sc-12919-R); mouse polyclonal anti-p-ERK1/2, (1∶200, Cell signaling Technology, 5726, Beverly, MA, USA), then sections were washed and incubated for 1 h with fluorescence-labeled secondary antibodies (goat anti- rabbit; Goat anti-Mouse) at room temperature. The double-stained images were examined using Leica DFC500 (Bioscience).

### Cdk5 kinase activity assay

Cdk5 kinase assay was performed as described previously [28], [29]. Briefly, tissue lysates was homogenized in immunoprecipitation buffer (50 mM HEPES, pH 7.5, 150 mM NaCl, 1 mM EDTA, 2.5 mM EGTA, 1 mM DTT, 1% Triton X-100, and protease inhibitor cocktail from Roche). Lysates were incubated on ice for 10 min, and then centrifuged at 10,000 g for 10 min at 4°C. Supernatant was collected, and protein concentrations were determined using the BCA assay kit (Pierce, Rockland, IL). Equal amounts (500 µg) of protein were incubated with 2 µg of anti-Cdk5 antibody at 4°C for 3 h. Protein A-Sepharose beads (Santa cruz) was added to the samples and incubation was continued for a further 12 h, after which samples were washed four times with assay buffer (20 mM Tris–HCl (pH 7.5), 20 mM MgCl_2_, 1 mM EDTA, 1 mM EGTA and 0.1 mM dithiothreitol). To determine kinase activity of Cdk5, the corresponding immunoprecipitates were incubated with 6 ml assay dilution buffer containing 10 µg histone H1 (14–155; Upstate) and 12 ml magnesium/ATP mixture (20–113; Upstate) in a final volume of 30 ml at 30°C for 30 min. The reaction was terminated by the addition of SDS sample buffer, followed by boiled for 5 min. Proteins were separated by 12% SDS-PAGE gels and transferred to PVDF membranes. Histone H1 and p-Histone H1 levels were detected, and the phosphorylation level of Histone H1 was indicated as the Cdk5 activity.

### Statistical analyses

We quantified the Western blots in two steps, first, using β-actin levels to normalize (e.g., determine the ratio of p-ERK1/2 to β-Actin amount) protein levels and control for loading differences in the total protein amount. Second, we presented protein level changes in treatment groups as a percentage of those naive groups. 100% of protein level changes refer to control levels for the purpose of comparison to experimental conditions. Data are expressed as means ± SEM. Differences between groups were analyzed using either the Student's t test or one-way ANOVA. All the data of rat behavioral experiments are expressed as means ± SD. Statistical analyses were performed by two-way repeated-measures ANOVA followed by Bonferroni's multiple comparison tests. The standard for statistical significance was p<0.05.

## Results

### Peripheral CFA-induced inflammation increased ERK activation and Cdk5 phosphorylation in SCDH

**Figure 1:**
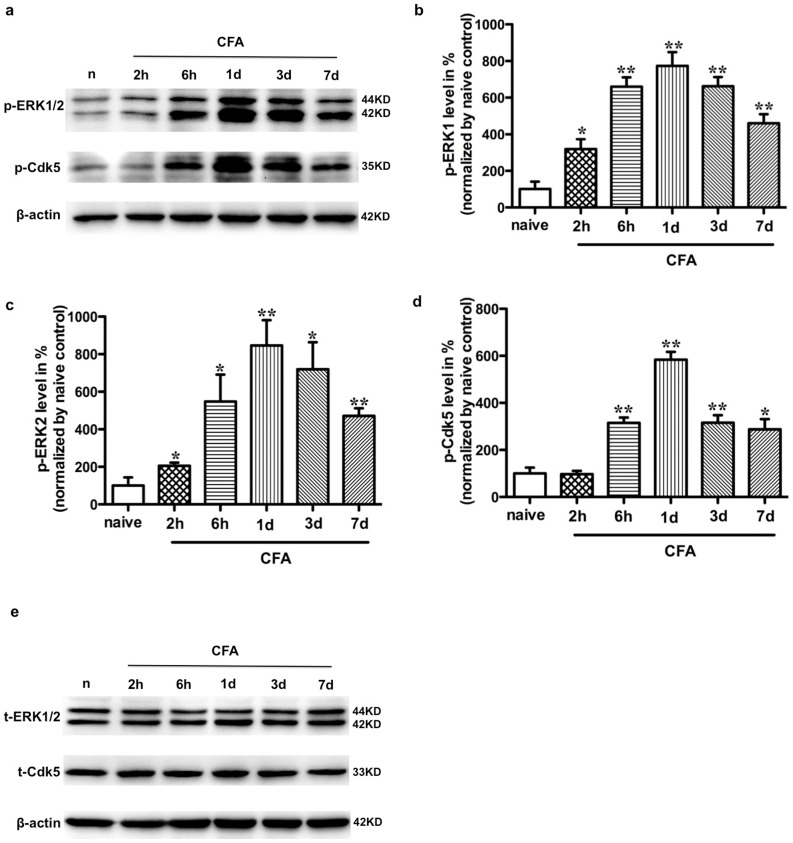
CFA injection increases the levels of p-ERK and p-Cdk5^S159^ expression in SCDH. a. Time course of ERK1/2 activity (panel 1) and p-Cdk5^S159^expression (panel 2) from 2 h to 1 week after CFA injection in rats as compared to naïve condition (lane1) in the Western blot analysis. b. Quantification of ERK1 activity in the dorsal horn. Columns represent means ± SEM. Data were normalized to naïve control and ANOVA followed by Dunnett's Multiple Comparison Test. *P<0.05, **P<0.01, as compared with naive rats. c. Quantification of ERK2 activity in the dorsal horn. d. Quantification of the levels in p-Cdk5^S159^ in the dorsal horn. e. CFA injection does not affect total ERK1/2 and total Cdk5 expression in SCDH compared to naïve control (lane 1) in the Western blot analysis. N = 3.

To investigate the changes in protein phosphorylation levels of ERK and Cdk5 in the SCDH after peripheral persistent inflammation, we injected 100 µl CFA into the plantar surface of a hindpaw. ERK activation by CFA was confirmed by Western blot analysis. In agreement with previous studies [23], [30], the phosphorylation levels of both ERK1 (Fig. 1a, b) and ERK2 (Fig. 1a,c) were very weak in naïve rats, began to increase at 2 h, reached a peak at 1 day, and remained at a high level for up to 1 week in the ipsilateral dorsal horn after CFA injection. Previous studies have shown that Cdk5 was markedly activated by peripheral noxious stimuli [9], [20], [31], phosphorylation of Cdk5 at the site of Ser159 residue (p-Cdk5^S159^) increases the enzymatic activity of Cdk5 activating kinases [13], [14], [15], we therefore assessed the effects of CFA induced peripheral noxious stimuli on the p-Cdk5^S159^ levels. Immunoblotting of p-Cdk5^S159^ showed a significantly time-dependent change, being elevated visibly at 6 h (3-fold), reaching a maximal level at 1 day (5.8-fold) and retaining a high level 1 week after CFA injection (Fig. 1a, d). The above results showed that ERK activation happened earlier (2 h) than Cdk5 activity (6 h) after CFA injection. The time course of p-Cdk5^S159^ expression was similar to the Cdk5 activity (4-fold at 6 h and 5-fold at 1 day) induced by CFA injection according to a previous report [9], which suggested that p-Cdk5^S159^ may be closely associated with Cdk5 activity.

### MEK inhibitor U0126 significantly mitigated heat hyperalgesia induced by CFA injection

**Figure 2:**
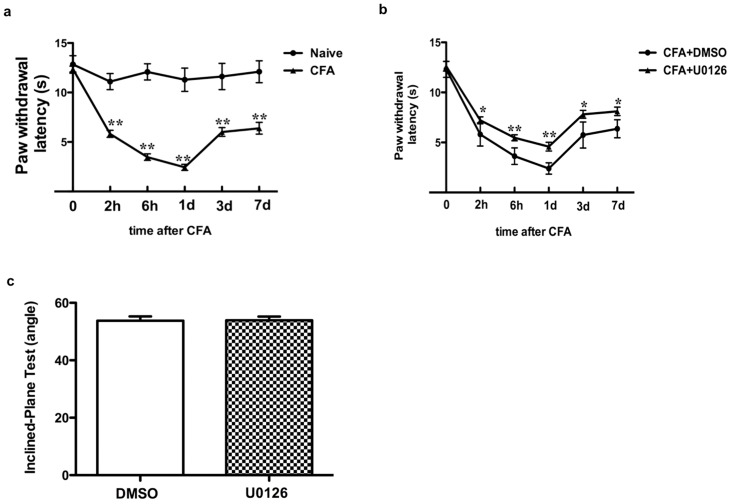
Intrathecal administration of U0126 attenuates CFA-induced heat hyperalgesia. a. PWLs to thermal stimuli significantly decreases after intraplantar injection of CFA; **p<0.01. b. Compared to the PWL of the control group treated with DMSO, PWL to thermal stimuli significantly increases following intrathecal injection of U0126 30 min before and once per day for 1 week after CFA injection. The symbols represent mean and vertical lines represented SD. Data were analyzed by two-way ANOVA with repeated measures followed by Bonferroni's multiple-comparison tests, *p<0.05, **p<0.01. c. 2 µg U0126 does not affect rat movement as detected by the inclined plane test before CFA administration. N = 6.

It is well known that nociceptive behavior associated with inflammatory pain is linked to heat hyperalgesia. We observed the changes in nociceptive behavior following CFA injection by measuring thermal PWLs. The average thermal PWL threshold (seconds) of ipsilateral hind paw was significantly reduced 2 h to 7 d post CFA injection (2 h, 5.82±0.15 s, 52.4%; 6 h, 3.8±0.09 s, 34.3%; 1 d, 2.44±0.12 s, 21.6%; 3 d, 6.02±0.12 s, 51.8%and 7 d, 6.38±0.09 s, 52.8%) compared to normal rats during the same time spans (2 h, 11.10±0.82 s; 6 h, 11.08±0.24 s; 1 d, 11.29±1.18 s; 3 d, 11.62±1.33 s and 7 d, 12.1±1.10 s, **p<0.01, Fig. 2a, n = 6). In addition, we tested the effects of a selective MEK inhibitor U0126 on the thermal PWLs, we intrathecally injected U0126 30 min before CFA administration and then once per day (for 7 days) after injection. Nociceptive responses were examined from 2 h to 1 week after CFA injection. Intrathecal injection of 2 µg U0126 did not affect rat movement before CFA injection as detected by the inclined plane test (Fig. 2c), however, it significantly increased thermal PWL threshold 2 h after CFA injection and lasted for 1 week (2 h, 7.22±0.85 s, 124%; 6 h, 5.57±0.65 s, 153%; 1 d, 4.58±1.06 s, 190%; 3 d, 7.81±0.97 s, 136% and 7 d, 8.11±1.04 s, 127%) compared to vehicle control (2 h, 5.81±1.16 s; 6 h, 3.63±0.82 s; 1 d, 2.4±0.57 s; 3 d, 5.75±1.3 s and 7 d, 6.37±0.90 s, *p<0.05, Fig. 2b, n = 6).

### MEK inhibitor U0126 attenuated increases in p-Cdk5^S159^ levels and Cdk5 activity in SCDH induced by CFA injection

**Figure 3:**
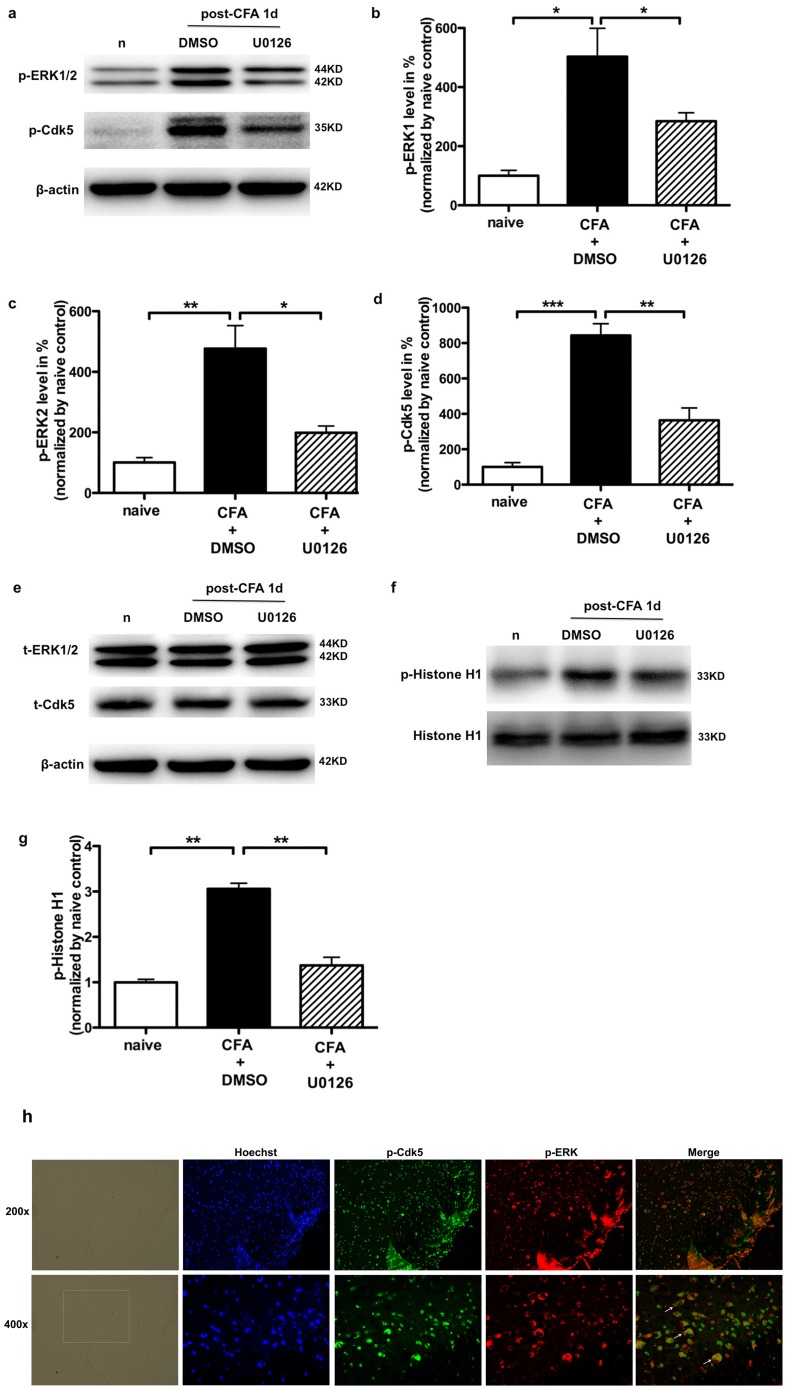
U0126 attenuates p-Cdk5^S159^ increase and Cdk5 activity in SCDH induced by CFA administration. a. U0126 mitigates CFA-induced p-ERK1/2 and p-Cdk5 increases in spinal cord in the Western blot analysis. b and c. Quantification of the Western blot shows that ERK1/2 activity by CFA is attenuated after U0126 treatment, *p<0.05, **p<0.01. d. Quantification of the Western blot shows that the increase of p-Cdk5^S159^ by CFA is attenuated after U0126 treatment, **p<0.01, ***p<0.001. e. U0126 treatment does not affect total-ERK1/2 and total-Cdk5 expression in spinal cord 1 d after CFA injection. f. U0126 mitigates CFA-induced Cdk5 kinase activity in spinal cord detected by an in vitro phosphorylation assay. Histone H1 was used as a substrate. g. Quantification of Cdk5 activity (density of phosphorylated H1 band) in the SCDH. Columns represent means ± SEM for three separate experiments. h. Double-immunofluorescence staining for p-ERK (red), p-Cdk5 (green), Hoechst [a cell nuclear marker (blue)] in ipsilateral spinal enlargement at 1 day after CFA injection. P-Cdk5^S159^ colocalizes with p-ERK (white arrow head).

To explore whether the elevation of p-Cdk5^S159^ levels following CFA (1 d) was mediated by an activation of ERK, MEK inhibitor U0126 2 µg was intrathecally administered twice (30 min before and 1 d after intraplantar CFA injection). As shown in Fig. 3, the protein levels of p-ERK1 and p-ERK2 were greatly enhanced to 503.44±55.36 (n = 3, p<0.05) and 446.56±44.07% (n = 3, p<0.01) respectively of naïve control following CFA (1 d). After U0126 administration, the protein levels were declined to 217.76±12.03% (n = 3, p<0.05, Fig. 3a, 3b) and 198.92±12.99% (n = 3, p<0.05, Fig. 3a, 3c) of naïve control, but still higher than naïve control. Meanwhile, the protein level of p-Cdk5^S159^ was remarkably increased to 842.62±38.66% (n = 3, p<0.001) of naïve following CFA (1 d), and then declined to 363.37±40.83% (n = 3, p<0.05) of naïve following U0126 injection (Fig. 3a, 3d). Moreover, We performed an in vitro phosphorylation assay and found that Cdk5 activity was relatively low in naïve rats, but there was a significant elevation of Cdk5 activity in the SCDH following CFA treatment (3.06±0.07, n = 3, p<0.01). After the treatment of U0126, the level of Cdk5 activity was decreased to 1.096±0.09 (n = 3, p<0.01).

It is well known that the distribution and subcellular localization in tissues can help identify the functions of the protein. Therefore, to test whether the p-ERK-positive neurons and p-Cdk5-expressing neurons belong to the same subset of dorsal horn cells, we performed double immunofluorescence for p-ERK and p-Cdk5^S159^. Almost all p-ERK positive neurons in the superficial dorsal horn also express p-Cdk5 1 d after CFA injection in the superficial dorsal horn of the lumbar enlargement (Figure 3e), indicated that p-Cdk5^S159^ may closely interact with p-ERK after peripheral inflammatory stimulation.

### Cdk5 inhibitor roscovitine suppressed heat hyperalgesia, but had no effect on increases of p-ERK levels by CFA-induced peripheral inflammation

**Figure 4:**
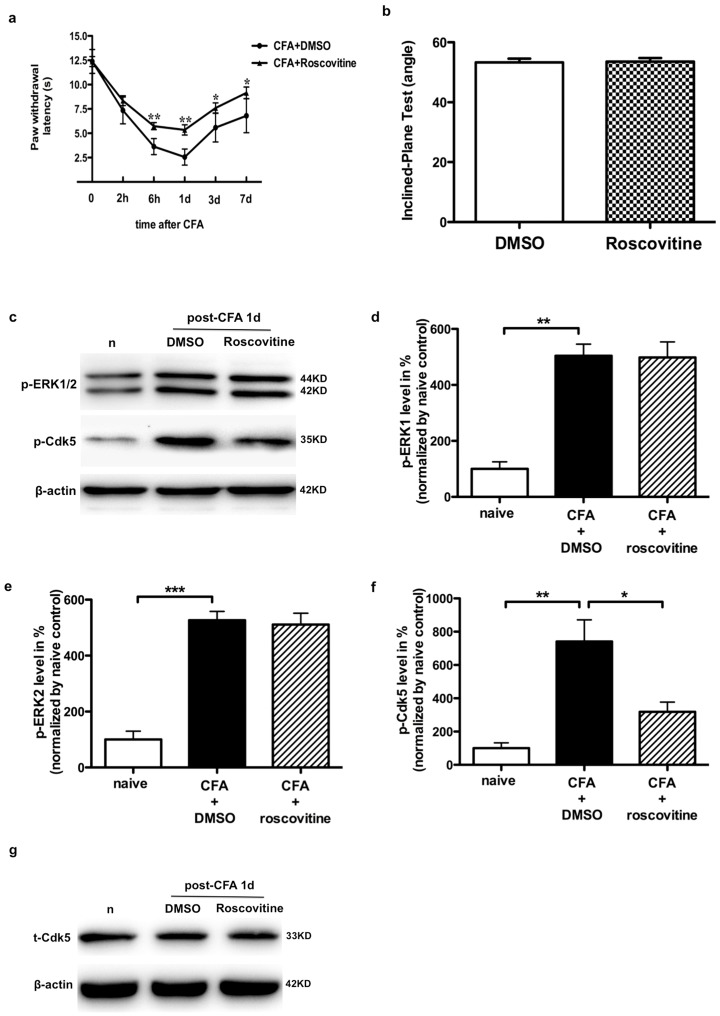
Roscovitine does not affect ERK1/2 activity in SCDH induced by CFA injection. a. Effects of roscovitine on the heat hyperalgesia compare to the DMSO treatment 2-way ANOVA with repeated measures followed by Bonferroni's multiple-comparison tests. *p<0.05, **p<0.01, n = 6. b. 100 µg roscovitine does not affect rat movement as detected by the inclined plane test before CFA administration, n = 6. c. Effects of roscovitine administration on ERK1/2 activity and p-Cdk5^S159^ expression after CFA in the Western blot analysis. d and e. Quantification of the Western blot shows that roscovitine treatment does not affect ERK1/2 activity by CFA, **p<0.01, n = 3. f. Quantification of the Western blot shows that the increase of p-Cdk5^S159^ by CFA is attenuated after roscovitine treatment, *p<0.05, **p<0.01, n = 3. g. Roscovitine treatment does not affect total-Cdk5 expression in spinal cord 1 d after CFA injection.

To further determine the role of Cdk5 phosphorylation in the CFA induced-inflammation. Cdk5 inhibitor roscovitine (100 µg) was intrathecally injected in rats (30 min before and once per day (for 7 days) after CFA injection). At the dose we used, we did not observe obvious toxicity of this inhibitor, animals behaved normally, and locomotion was unaffected before CFA injection (Fig. 4b). Heat hyperalgesia was significantly attenuated by roscovitine treatment when tested at 6 h to 7 d post CFA injection following CFA (Fig. 4a). Meanwhile, p-Cdk5^S159^ level was remarkably enhanced to 224.24±74.74% (n = 3, p<0.01) of naïve following CFA (1 d), and then reduced to 318.60±33.55% (n = 3, p<0.05) of naïve following roscovitine injection (Fig. 4c, f), suggested that p-Cdk5^S159^ expression in SCDH contributed to heat hyperalgesia by CFA injection. However, Immunoblotting of p-ERK showed that ERK activity induced by CFA did not blocked by roscovitine treatment (Fig. 4c, d, e), indicated that ERK activity induced by persistent inflammatory was not regulated by Cdk5 phosphorylation in SCDH after CFA inflammatory stimulation.

### U0126 reduced the numbers of spontaneous flinches, attenuated the elevation in p-Cdk5^S159^ levels and Cdk5 activity after formalin injection

**Figure 5:**
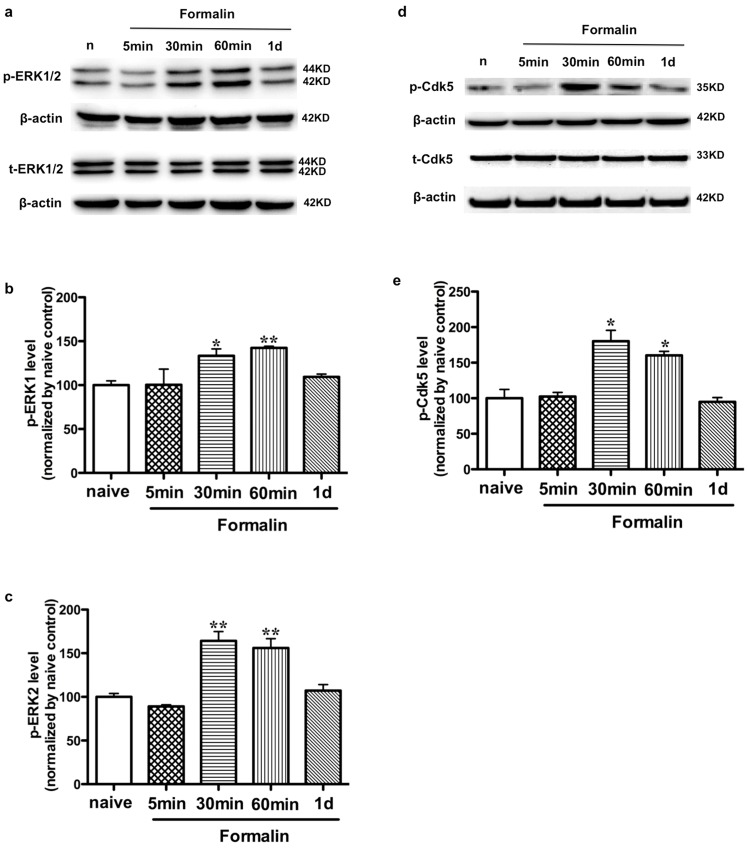
Levels of p-ERK and p-Cdk5^S159^ increase in SCDH after formalin injection. a. Time courses of p-ERK1/2 (panel 1) and t-ERK1/2 expression (panel 3) from 5 min to 1 d after formalin injection in rats as compared to naïve condition (lane1) in the Western blot analysis. b. Quantification of ERK1 activity in the dorsal horn. Columns represent means ± SEM. Data were normalized to naïve control and ANOVA followed by Dunnett's Multiple Comparison Test. *P<0.05, **P<0.01, as compared with naive rats. c. Quantification of ERK2 activity in the dorsal horn. d. Time courses of p-Cdk5^S159^ (panel 1) and t-Cdk5 (panel 3) expression from 5 min to 1 d after formalin injection in rats as compared to naïve condition (lane1) in the Western blot analysis. e. Quantification of levels in p-Cdk5^S159^ in the dorsal horn.

Plantar injection of formalin can produce an acute inflammatory pain [32], [33], [34], which displayed discrete biphasic behavioral responses consisting of an early short-lasting response (phase I, 0–10 minutes post-injection), followed by a late, prolonged response (phase II, approximately 10–60 minutes post-injection). Previous study reported that Cdk5 inhibitor roscovitine attenuated the formalin-induced flinch response [35]. Our study showed that compared to the naïve control, the levels in p-ERK (Fig. 5a, b, c) and p-Cdk5^S159^ (Fig. 5d, e) in SCDH were upregulated at 30 min and 60 min (phase II) after formalin injection, and the elevation was decreased at 1 d post-injection.

**Figure 6:**
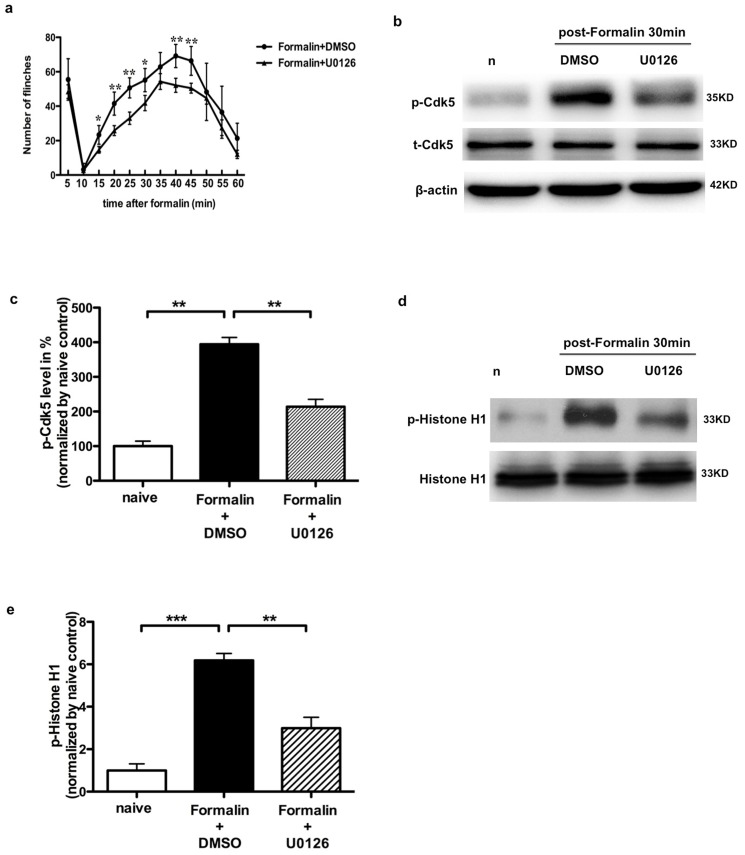
U0126 attenuates spontaneous nociceptive responses, the elevation of p-Cdk5^S159^ and Cdk5 activity in SCDH induced by formalin administration. a. U0126 treatment significantly decreases the numbers of spontaneous flinches induced by formalin injection in the 2^nd^ phase (10–45 min), but not in the 1^st^ phase (0–10 min). Data were analyzed by two-way ANOVA with repeated measures followed by Bonferroni's multiple-comparison tests. b. U0126 mitigates formalin-induced p-Cdk5 increase in spinal cord in the Western blot analysis. c. Quantification of the Western blot shows that the increase of p-Cdk5^S159^ by formalin is attenuated after U0126 treatment, **p<0.01. d. U0126 mitigates formalin-induced Cdk5 kinase activity in spinal cord detected by an in vitro phosphorylation assay. Histone H1 was used as a substrate. e. Quantification of Cdk5 activity (density of phosphorylated H1 band) in the SCDH, **p<0.01, ***p<0.001.

To determine whether the Cdk5 phosphorylation induced by formalin injection was regulated via ERK activity, 2 µg U0126 was intrathecally injected 30 min before formalin injection. We were able to show that U0126 attenuated the numbers of formalin-induced flinch responses during phase II (Fig. 6a, n = 6). We therefore assessed the effects of U0126 on the levels of p-Cdk5 and t-Cdk5 30 min after formalin injection. Immunoblotting of p-Cdk5 showed that there was visible reduction (214.16±12.25 versus 394.34±11.28) in the band representing p-Cdk5 in the SCDH compared to the vehicle control (n = 3, p<0.01, Fig. 6b, c). There was no significant difference in the levels of t-Cdk5 among the various treatments. In addition, the level of Cdk5 activity was remarkably increased to 6.18±0.32 (n = 3, p<0.0001) of naïve following formalin (30 min), and then declined to 2.99±0.51 (n = 3, p<0.001) of naïve following U0126 injection. These findings suggested that the formalin-induced acute inflammatory pain, phosphorylation of Cdk5 at Ser159 site and Cdk5 activity were dependent on the ERK activity in the SCDH.

## Discussion

Cdk5 was recently shown to be a key regulator in inflammatory induced pain hypersensitivity [10], [11], [12], [36]. Both p35 and p25 proteins are established positive regulator of Cdk5 activity. Previous studies reported that p35 and p25 protein levels increased as well as Cdk5 activity, with a highly positive linear correlation with nociceptive responses induced by inflammation in rats [9], p35 gene knockout and transgenic mice overexpressing were important for the thermal response in mice [10]. Ser-159 phosphorylation of Cdk5 is directly associated with Cdk5 activity [14], [16]. As shown in Fig. 1, peripheral persistent inflammation induced, after a short latency, a persistent Ser159 phosphorylation of Cdk5 in ipsilateral SCDH, without any significant change in the protein levels of total Cdk5. Inhibition of this activation, using an Cdk5 inhibitor roscovitine, blocked elevation of p-Cdk5^S159^ expression in this particular subset of dorsal horn, as well as reduced inflammatory pain hypersensitivity, implied that Cdk5 phosphorylation at ser159 site plays an important role in the development of inflammatory pain. Although roscovitine significantly inhibited Cdc2, Cdk2, Cdk5 [37], roscovitine mainly inhibited Cdk5 activity, Cdk5 was primarily confined to the postmitotic neurons, and Cdc2 and Cdk2 were expressed only at the embryonic stage [38]. All Cdk kinase activities were silenced in mature neurons except Cdk5 [39], [40].

However, it is still unclear how Cdk5 phosphorylation is regulated during inflammation-induced pain signaling. It is unknown which modulators that are triggered by inflammation affect Cdk5 phosphorylation. ERK is important for intracellular signal transduction and play critical roles in regulating inflammatory responses [23], [41], [42]. ERK is required for noxious stimulation-induced phosphorylation of the NMDA receptor NR1 subunit [43] and activity-dependent insertion of AMPA. Moreover [44], ERK activation is essential for CREB phosphorylation, prodynorphin and NK-1 in SCDH neurons after noxious stimulation [23], [45]. Previous studies reported that TNF-α receptor/Erk/Egr1 signal pathway could mediate p35 up-regulation and increase Cdk5 activity in PC12 cells. TNF-α is an established proinflammatory cytokine and also induced by CFA inflammation, thus it is conceivable that the CFA-induced inflammation may elevate the levels of p-Cdk5^S159^ by increasing ERK activity. This hypothesis was further supported by the findings that intrathecal administration of a potent and selective MEK inhibitor U0126 attenuated heat hyperalgesia and ERK activity, as well as the increase of p-Cdk5^S159^ expression induced by CFA inflammation. In addition, p-Cdk5^S159^ was induced by CFA in the same cells of dorsal horn that express p-ERK. It is reported that both of Cdk5 and ERK are associated with complexes of cytoskeletal proteins in rat brain, fractions enriched in ERK kinase activity also exhibited co-elution of Cdk5 [46], implying Cdk5 is closely associated with ERK. In a previous study we found that Cdk5 is mostly localized in the cytoplasm of spinal cord neurons in naïve rats [31], in this study, we found that p-Cdk5^S159^ was mainly in the cellular nucleus after CFA injection, meanwhile, p-ERK was mainly in the cytoplasm and lightly in the nucleus, which is fully consistent with a previous study [23] demonstrating that the CFA-induced p-ERK was found only in neurons and could be observed in the nucleus. These results pointed to a possible transcriptional role for the interaction between ERK and Cdk5 activated kinase. Studies in vitro showed that TNF-α induces sustained increase in the levels of p35 protein and promoter through activation of the ERK1/2 pathway, thereby increasing Cdk5 kinase activity. U0126 significantly decreased p35 promoter activity induced by TNFα [47]. However, intraplantar injection of CFA or carrageenan in rats induced inflammatory significantly elevated the level in p25 protein, other than p35 in SCDH [9], [10]. Studies on PC12 cells showed that Cdk5/p25 activity is modulated by an unidentified kinase that phosphorylates Cdk5 at Ser159 and enhances its kinase activity, a Ser (159)-to-Ala (S159A) cdk5 mutant did not show similar activation [48]. Our results showed that Cdk5 was phosphorylated at Ser159 response to peripheral inflammation in rats. Collectively, we postulate that CFA-induced ERK activity mediated Cdk5 phosphorylation at ser159 site possibly via the up-regulation of p25 promoter in SCDH. Using situ hybridization, Pareek et al proved that Cdk5 and p35 were expressed strongly in neurons of the DRG but were expressed at a much lower level in neurons of the SC. But the cdk5 activity was stronger in SC than DRG [10]. However, using western blot analysis, a previous study reported that the expression levels of Cdk5 and p35 were higher in the dorsal horn than in DRG, and p25 could only be detected in the dorsal horn [9]. Unlike the predominantly peripheral plasma membrane association exhibited by p35, p25 lacks the myristoylation sequence for membrane targeting and is therefore not associated with the plasma membrane. Conceivably, p25 may overactivate and remove Cdk5 from its normal compartments and locate in the perinuclear region and nuclei [49]. Previous studies have reported that the cleavage of p35 to p25 results in the translocation of Cdk5/p25 complexes to the nucleus, increasing histone H1 kinase activity [50]. Cdk5 protein predominantly expressed in cytoplasm near the membrane, Cdk5 activation was a switch in the profile of Cdk5 immunostaining from predominantly cytoplasmic to a more diffuse staining in both the cytoplasm and nucleus [47]. Our study found p-Cdk5 was mainly expressed in the nucleus in SCDH after CFA injection, so we postulate the increase of p-Cdk5^S159^ is critical for Cdk5 activity in the inflammatory model and p25 is necessary for this process.

Additionally, in the present experiments, we found that the level of p- Cdk5^S159^ increased in the 2^nd^ phase of formalin-induced acute inflammatory pain, moreover, the elevation could be attenuated by U0126 intrathecal administration. These findings further supported the results that p-Cdk5^S159^ participated in the development of peripheral inflammatory pain, which was regulated by ERK activity.

Yang et al have reported that Cdk5 activity was upregulated in dorsal horn neurons, which was detected by an in vitro phosphorylation assay and Histone H1 was used as a substrate. Their studies have shown that intrathecal pretreatment with Roscovitine, a specific inhibitor of Cdk5 activity, and intrathecal delivery of the DN-Cdk5 (N144) gene or overexpression of Cdk5, p35 or p25 in dorsal horn neurons significantly alleviated or enhanced heat hyperalgesia, but not mechanical allodynia. Pretreatment with Roscovitine have no effect on p-ERK after CFA injection 15 min and 6 h [9]. Our study also found that intrathecal pretreatment with Roscovitine could alleviate heat hyperalgesia induced by CFA injection, and didn't affect the level of p-ERK in spinal cord. The differences between the two studies: 1) our study found the level of p-Cdk5^S159^ was upregulated in dorsal horn induced by CFA or formalin injection. 2) Most p-Cdk5^S159^ colocalized with p-ERK in spinal cord dorsal horn 1 d after CFA injection, and Intrathecal pretreatment with ERK inhibitor U0126 significant decrease p-Cdk5^S159^ expression, meanwhile, U0126 also inhibit Cdk5 activity after CFA or formalin injection. The focus of our work was on the phosphorylation of Cdk5 itself in inflammatory pain. Previous studies reported that the acute stimulation of opioid receptors leads to upregulation of Cdk5 activation through a MEK-ERK -dependent pathway, and to disruption of MEK-ERK signalling by a cdk5/p35-induced MEK1 inhibition [51]. Consistently, our studies found that peripheral inflammatory stimulation resulted Cdk5 phosphorylation via ERK activity. However, Cdk5 inhibitor roscovitine has no effect on ERK1/2 activity after CFA injection.

## Conclusions

In this study, MEK inhibitor U0126 attenuated the inflammatory nociceptive response and the activation of spinal ERK 1/2 and Cdk5 phosphorylation in the CFA or formalin-induced inflammatory pain. These results strongly suggest that Cdk5 phosphorylation at ser159 is an important downstream of ERK activity, contributing to Cdk5 activity in the early stage of peripheral inflammatory pain. Thus, in the clinic, p-Cdk5^S159^ would be an advantageous target for the control of chronic pain development to prevent or treat pain.
